# Scaling up X-ray holographic nanotomography for neuronal tissue imaging

**DOI:** 10.1364/BOE.559263

**Published:** 2025-04-25

**Authors:** Jayde Livingstone, Carles Bosch, Aaron T. Kuan, Lucas Benoït, Paolo Busca, Thierry Martin, Mohd F. E. B. Mazri, Wangchu Xiang, Wei-Chung Allen Lee, Andreas T. Schaefer, Peter Cloetens, Alexandra Pacureanu

**Affiliations:** 1 ESRF, The European Synchrotron, Grenoble, France; 2 Present address: Univ. Grenoble Alpes, CNRS, Grenoble INP, LPSC-IN2P3, 38000 Grenoble, France; 3 Sensory Circuits and Neurotechnology Laboratory, The Francis Crick Institute, London, United Kingdom; 4 Department of Neurobiology, Harvard Medical School, Boston, MA, USA; 5 Present address: Department of Neuroscience, Yale School of Medicine, New Haven, CT, USA; 6 F. M. Kirby Neurobiology Center, Boston Children’s Hospital, Boston, MA, USA; 7 Department of Neuroscience, Physiology and Pharmacology, University College, London, UK

## Abstract

Neuronal circuit reconstruction from X-ray holographic nanotomography (XNH) images of neuronal tissue requires overcoming limits in acquisition speed, image quality, and sample size. To fully exploit the higher brilliance of the European Synchrotron’s upgraded source, advances in endstation instrumentation and adapted data collection strategies are necessary. A detector upgrade combined with continuous scanning for XNH of neural tissue samples at the ESRF’s ID16A beamline demonstrates preserved or improved quality of images of large samples whilst increasing data acquisition time by more than a factor of two. This is a critical step in enabling the scaling up of XNH for neuronal tissue imaging.

## 1. Introduction

The precise mapping of neural connectivity and its correlation with neurological functions [1–4] is a prerequisite to understanding brain functions and neurological disorders [2,5–11]. Reconstructing entire neural circuits and mapping their densely packed, sub-100 nm diameter [1,12] synaptic connections requires high-resolution, multi-scale imaging.

The current gold standard imaging technique for neural circuit reconstruction is electron microscopy (EM), capable of resolving individual synapses in small neural circuits [13]. EM has been applied to entire brain mapping of the nematode *C. elegans* (10^2^ neurons) [14], fruit fly larvae (10^3^ neurons) [15,16], the adult fruit fly (10^5^ neurons) [17–19] and larval zebrafish (10^5^ neurons) [20,21]. The destructive nature of serial sectioning required for EM inevitably leads to loss of information and geometric distortions which complicate neuron-tracing [1] and the scaling up to entire mammalian brains. Mapping the entire mouse brain, for example, with a volume of ∼ 450^3^ [22] and 10^8^ neurons at synaptic resolution using EM is estimated to take several years, with manual tracing requiring orders of magnitude longer [23]. So far, efforts to map neural circuits in the mouse brain have been limited to volumes of 1.5 mm^3^ or less.

Synchrotron-based hard X-ray phase contrast imaging is emerging as a promising approach for accelerating neural imaging in a non-destructive manner [12,24–26]. X-ray holographic nanotomography (XNH) [12,27–31] is a coherent imaging technique offering full-field imaging at spatial resolutions in the range of tens of nanometers using a focused nanoprobe, and has demonstrated its potential for nanoimaging of neural tissues via comparative studies with EM [30,32,33] and histology [12,34]. The non-destructive nature of XNH makes it compatible with complementary examinations, including post-hoc EM [30]. XNH is one of the nanoimaging techniques offered by the ID16A beamline at the European Synchrotron (ESRF), Grenoble, France. To fully benefit from the ESRF’s fourth generation Extremely Brilliant Source (EBS) [35] and to address the need of scaling up nanoimaging of neuronal tissue, we propose a strategy combining instrumentation, data collection and data reconstruction developments.

## 2. Materials and methods

### 2.1. X-ray holographic nanotomography of neural tissues

The ID16A beamline at the ESRF produces high brilliance, nanofocused, coherent x-rays with energies of 17.1 or 33.3 keV for nanoimaging applications. X-ray holographic nanotomography (XNH) is one of the nanoimaging techniques offered by ID16A. The endstation on which XNH is performed consists of a vacuum chamber evacuated to $1.0\times 10^{-7}$ mbar and cryogenically cooled. The samples are placed on a high precision rotational stage [36]. XNH consists of acquiring 2000 projections over 180^∘^ at four different propagation (or defocusing) distances [27], which are set depending on the desired effective pixel size. An example for 90 *μ *m pixels is given in section 2.4. Projections are typically acquired in a “step-by-step” mode to allow for suppression of ring artifacts based on random sample displacements between successive projections [37]. After traversing the specimen, the beam propagates freely over a distance of approximately 1.2 m to the detector, which is composed of a scintillator lens-coupled (10× objective) to a camera. More details about the detectors used in this study are provided in section 2.2.

In order to evaluate the performance of the new detector and data collection techniques, XNH data were acquired on mouse tissue samples from the spinal cord, the olfactory bulb and the cerebral cortex. The samples were prepared using EM protocols, including staining with osmium tetroxide, and embedding in EPON resin [38]. The spinal cord and cortex tissues were cut with a ceramic blade into rectangular rods, while the olfactory bulb tissue was cut into rounded pillars using a femtosecond laser (515 nm, <350 fs pulse duration, 100 kHz pulse frequency and laser power of 20%) [39].

Phase maps were retrieved from the raw XNH data using a holographic reconstruction based on an initial estimate from a multi-distance Paganin algorithm followed by an iterative refinement by a non-linear conjugate gradient method, using a fixed number of iterations (10) which corresponds to plateauing of the minimisation of the error function [40]. Tomographic reconstructions, using the phase maps as input, were performed using one of two software available at the ESRF; PyHST2, a distributed tomographic reconstruction code that offers filtered back-projection and iterative techniques [41], or Nabu, a recently released tomographic reconstruction code offering filtered back-projection and iterative techniques (https://gitlab.esrf.fr/tomotools/nabu) as well as post-processing options such as ring removal, including the combined wavelet and Fourier analysis-based algorithm proposed by Münch *et al.* [42]. In this work, all reconstructions were performed using filtered back-projection.

### 2.2. Imaging detector upgrade

The first step in scaling up nanoimaging of neural tissues requires upgrading beamline instrumentation, or more specifically, replacing the existing CCD-based FReLoN camera (ESRF, Grenoble, France) with a faster camera. We investigated the CMOS-based Ximea MX377 camera (Ximea GmbH, Münster, Germany) as a potential replacement. The full specifications of the two cameras are given in Table 1. The most important difference to note is the faster frame rate and readout time of the Ximea MX377 camera, both 10×
 faster than those of the FReLoN camera.

**Table 1.** Specifications of the ESRF FReLoN and Ximea MX377 cameras investigated during this study.

	ESRF FReLoN	Ximea MX377^*a*^
**Area (pixels^2^)**	4096 (H) × 4096 (V)	6144 (H) × 6144 (V)
**Pixel size**	15 *μ *m	10 *μ *m
**Active area**	61.44 × 61.44 mm^2^	61.44 × 61.44 mm^2^
**Sensor**	e2v CCD230-84 CCD^*b*^^,^^*c*^	Gpixel GSENSE6060 CMOS^*d*^^,^^*e*^
**Readout noise**	17.8 e^− ^ rms	5.17 e^− ^
**Full well capacity**	141000 e^− ^	105004 e^− ^
**Peak quantum efficiency**	93% (at 615 nm)	70% (at 550 nm)
**Resolution**	16 bit	16 bit
**Dynamic Range**	12.96 bit	14.3 bit
**Frame rate**	0.8 – 1.3 fps	16 fps
**Readout time**	750 ms	62 ms
**Shutter mode**	Global	Rolling
**Scintillator crystal** ^*f*^	Ga_3_Gd_5_O_12_:Eu (23 μ m thick)	Lu_2_SiO_5_:Tb (20 μ m thick)

^*a*^ Engineering grade, having either a total of >1500 defective pixels, >10 defective columns/rows, or >3 adjacent defective columns/rows [43]

^*b*^ e2v Technologies Ltd., Chelmsford, United Kingdom

^*c*^ Back-side illuminated

^*d*^ Gpixel Microelectronics Inc., Changchun, China

^*e*^ Front-side illuminated

^*f*^ See Wollesen *et al.* 2022 [44] and Wollesen *et al.* 2024 [45] for more details

The performance of the cameras was compared in terms of acquisition time using XNH datasets of the mouse spinal cord sample, acquired using an x-ray energy of 17.1 keV, 90 nm voxel size and reconstructed using PyHST2. The total time is the sum of acquisition times measured for each tomographic acquisition, and does not include the time for motor translations along the beam axis. As it is essential that image quality is not compromised at the expense of acquisition time, the cameras were also evaluated in terms of two common image quality metrics, spatial resolution and contrast-to-noise ratio (CNR), which are described in detail in section 2.5. Two different exposure times were used: a typical exposure time of 200 ms, and an “under-exposure" time of 70 ms.

### 2.3. Effect of sample size and tomographic acquisition mode on image quality

Imaging larger samples is inherent to scaling up XNH for neural tissue imaging. Image quality as a function of sample size was therefore evaluated, using XNH datasets of mouse olfactory bulb tissue. Two samples with diameters of 300 *μ *m and 800 *μ *m diameter (in the beam direction) were used for this study, and the image quality was quantified in terms of noise, as described in section 2.5.

In both cases the sample is larger than the detector field-of-view (FOV), and interior tomography artifacts are expected. Interior tomography causes structured noise resulting from contributions of sample structures outside the field-of-view, and is particularly strong in the case of neuronal tissue due to the intrinsic density and complexity of the architecture. Since pushing the sample size further beyond the detector FOV is expected to degrade image quality, we investigated the potential role of the tomography acquisition mode. For the larger sample, the quality of images acquired in a continuous scanning mode were compared to that of images acquired using the current standard; step-by-step (SBS) acquisition incorporating random-motion-based ring suppression. Continuous scanning offers the obvious advantage of being faster compared to SBS acquisition; however, random-motion-based ring suppression is not compatible with this mode. Post-processing ring removal has been investigated as an alternative option.

The different samples and acquisition modes are summarised in Table 2. All datasets were acquired using an x-ray energy of 33.3 keV, a voxel size of 60 nm and, unless otherwise indicated, an exposure time of 350 ms and 2000 projections.

**Table 2.** The different samples and XNH acquisition parameters used in this study.

Sample diameter (*μ *m)	Camera	Acquisition mode
300	FReLoN	Step-by-step^*a*^
800	FReLoN	Step-by-step
800	Ximea	Step-by-step
800	Ximea	Continuous

^*a*^ 330 ms exposure time, 1900 projections

All datasets were reconstructed using Nabu. For the dataset acquired in continuous mode, two reconstructions were performed: one without and one with ring correction (Münch).

Noise and contrast-to-noise ratios were evaluated as described in section 2.5 to compare image quality for different-sized samples and different tomographic acquisition and reconstruction modes. The acquisition time was also evaluated and compared for the different tomographic acquisition modes. As described in section 2.2, the acquisition time stated does not include the time for motor translations along the beam axis, it does however in the case of SBS acquisition, include the time required to perform the random translations between projections used for ring suppression.

### 2.4. Effect of holographic acquisition mode on image quality

The final strategy that was tested to reduce the acquisition time while maintaining all other tomographic imaging parameters (number of projections, exposure time, voxel size) is the acquisition of projections in fewer sample positions. Less data reduces the acquisition time, but also the information given as input to the phase retrieval algorithm and is therefore expected to degrade image quality. For this study, XNH datasets of mouse cortex tissue were acquired in continuous mode using 17.1 keV X-ray energy, 90 nm voxel size, 150 ms exposure time and reconstructed using Nabu with Münch ring correction. Acquisition was performed using the four distances typically used (referred to as D1, D2, D3 and D4), but the phase retrieval was performed twice, once on the entire dateset, and a second time using only distances D1, D3 and D4. The distances of D1, D2, D3 and D4 from the focal spot are given in Table 3. The choice of such a subset of sample positions allows the voxel size and field-of-view to be maintained. Tomographic reconstruction was then performed on both datasets using the Nabu software and Münch *et al.* [42] ring removal.

**Table 3.** Sample positions and their distances from the focal spot.

Sample position	Distance from the focal spot (mm)
D1	28.313
D2	29.848
D3	35.990
D4	48.743

Image noise was evaluated to compare image quality for the two holographic acquisition modes. The acquisition time was also evaluated.

### 2.5. Image quality metrics

This section describes the metrics that were used to evaluate image quality.

#### 2.5.1. Spatial resolution

Spatial resolution was evaluated using Fourier shell correlation (FSC), a standard quality measure in EM [46] which has seen increasing use for other imaging modalities [47–49]. FSC compares two independent half-datasets (in this case, two sub-volumes of the same dataset, one reconstructed from the odd-numbered projections and the other from the even-numbered projections) in a set of concentric shells in the Fourier space. The correlation of the half-datasets in each shell, evaluated for shell *i* using Eq. (1), is plotted as a function of spatial frequency and ranges from 1 (perfect correlation) at the lowest spatial frequencies to 0 (no correlation) at the highest spatial frequencies [46,50,51].

(1) FSC(ri)=Σ
r∈
riF1(r)⋅
F2(r)∗
Σ
r∈
riF12(r)⋅
Σ
r∈
riF22(r)

where $F_{1}(r)$ and $F_{2}(r)$ denote the complex-valued Fourier transform at spatial frequency r of volumes 1 and 2 respectively and ∗ signifies complex conjugation.

The intersection of the resulting curve with a predefined resolution criterion, in this case the half-bit threshold, [46], gives the inverse of the spatial resolution.

This method of evaluating spatial resolution is not appropriate when low spatial frequency artifacts, such as rings, are present in the dataset.

#### 2.5.2. Noise and contrast-to-noise ratio

Image noise, which can be defined as the random variation in pixel values and assumed to be independent of variations in tissue composition within the sample, can be quantified by evaluating the standard deviation of pixel values in the image background (region void of the sample). Given the small FOV compared to the sample size, XNH datasets often contain no data outside the sample. We have taken blood vessels, which are void of material except for the resin used to fix the sample, to represent the background.

If σ
background represents the standard deviation of background pixel values (and thus the image noise), the contrast-to-noise ratio (CNR) for a tissue of interest is given by:

(2) CNR=μ
tissue−
μ
backgroundσ
background

Given the need of tracing axons, either manually or automatically, in neural circuit reconstruction, we calculated the CNR for the heavily-absorbing myelin sheath surrounding axons. The values of μ
 and σ
 in myelin and the background were obtained for a number (variable, depending on the study) of regions of interest in the reconstructed datasets.

## 3. Results

### 3.1. Imaging detector upgrade

**Fig. 1.**
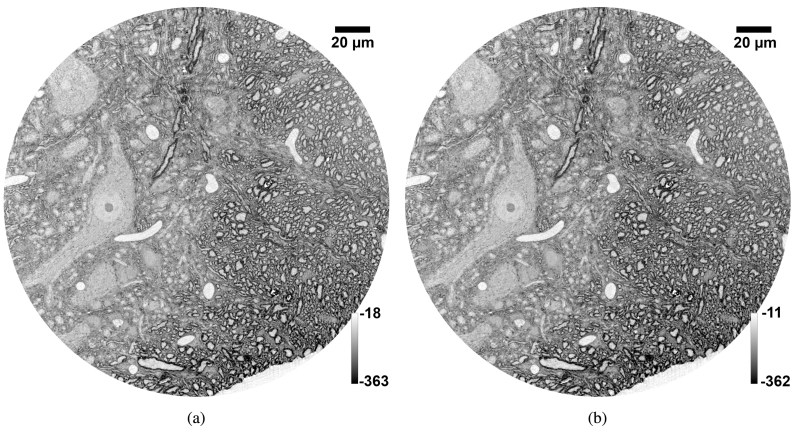
XNH images of a mouse spinal cord sample were acquired using a 90 μ m voxel size, 200 ms exposure time and beam energy of 17.1 keV. Corresponding slices of the datasets obtained with the FReLoN and Ximea MX377 cameras are shown in (a) and (b) respectively. The grey values are linked to the phase and hence the δ
 coefficient of the complex refractive index. Note that the 3D image quality is preserved, with FSC resolution measurements nearly identical, while the image acquisition time per frame is accelerated by a factor 3.6 when using the sCMOS-based camera, for the same exposure time of 200 ms per frame.

An example of corresponding slices of datasets acquired using the FReLoN and MX377 cameras with a 200 ms exposure time is shown in Fig. 1.

**Fig. 2.**
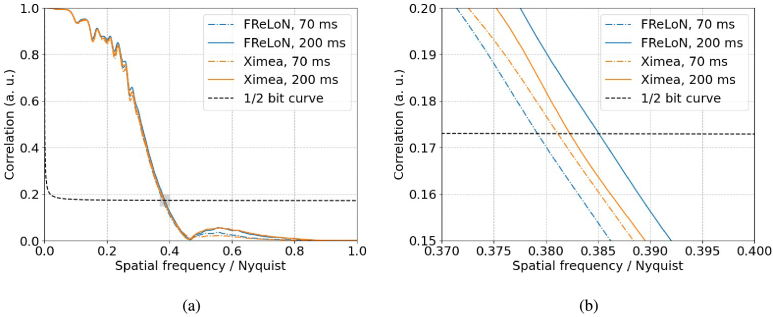
XNH images of a mouse spinal cord sample were acquired using a 90 μ m voxel size, 200 ms exposure time and beam energy of 17.1 keV. The spatial resolution of the FReLoN and MX377 cameras was evaluated using the intersection of Fourier shell correlation curves and the half-bit information threshold for 70 and 200 ms exposure times. The FSC plot in (b) corresponds to the shaded area in (a). The values of spatial resolution are between 234 and 237 nm, or approximately 2.6 pixels.

Figure 2(a) shows the FSC analyses for the FReLoN and Ximea MX377 cameras for 70 and 200 ms exposure times. The correlation has been plotted as a function of spatial frequency / Nyquist, where Nyquist corresponds to the Nyquist frequency ( $2\times f_{s}$ , where $f_{s}$ , the sampling frequency, is the inverse of the pixel size). The spatial resolution is thus given by the pixel size (90 nm) divided by the spatial frequency at which the FSC intersects the half-bit threshold.

The spatial resolution and CNR between myelin and the background measured for each camera at each exposure time are detailed in Table 4. The measurement time (not including time to change position between sample positions) and acquisition time per frame are also given. The 3D image quality is preserved, with FSC resolution measurements nearly identical, while the image acquisition time per frame is accelerated by a factor 3.6 when using the sCMOS-based camera, for the same exposure time of 200 ms per frame.

**Table 4.** Spatial resolution, CNR values and acquisition times measured for each camera and for each exposure time.

Camera	Exposure time (ms)	FSC intersection	Spatial resolution (nm)	CNR	Measurement time^*a*^ (min)	Acquisition time per frame^*b*^ (s)
FReLoN	200	0.382	234 (2.6 pixels)	13.3	173	0.950
FReLoN	70	0.379	237 (2.6 pixels)	11.5	154	0.820
MX377	200	0.382	235 (2.6 pixels)	11.4	156	0.262
MX377	70	0.381	236 (2.6 pixels)	9.6	136	0.132

^*a*^ The total time for an XNH acquisition for four sample positions including sample rotation and random displacements for ring artifact suppression, but excluding the sample displacement along the beam axis

^*b*^ The time required for a single projection (exposure time + detector readout), which is uniquely detector-dependent

### 3.2. Effect of sample size and tomographic acquisition mode on image quality

**Fig. 3.**
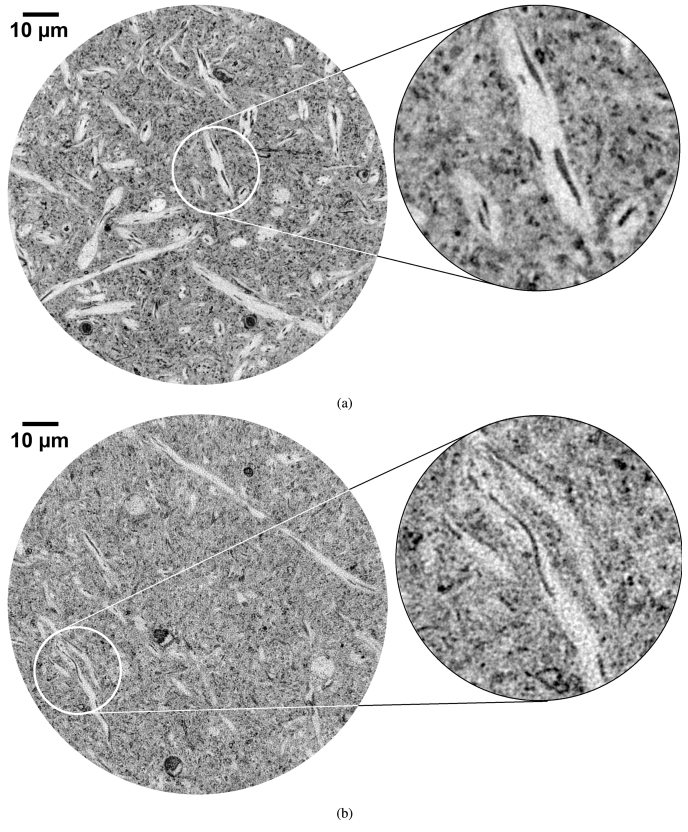
XNH images of mouse olfactory bulb tissue with diameters of (a) 300 μ m and (b) 800 μ m. The voxel size is 60 nm in both cases. The right hand side is a 4.5× zoom of the region-of-interest drawn in white on the left hand side. Both images were acquired using the FReLoN camera. The structured noise present in the large diameter sample comes from interior tomography effects, when the field-of-view of the acquisition is much smaller than the sample size. The unwanted contributions of the sample structures present outside the FOV are particularly strong in the case of neuronal tissue due to the intrinsic density and complexity of the architecture.

Figure 3 compares slices from reconstructed images acquired of the 300 μ m and 800 μ m diameter samples.

Interior tomography artifacts, visible as grainy or structured noise, are present, particularly for the larger sample. The image noise was evaluated as explained in section 2.5, giving σ
300μ
m=9.6±
0.1 and σ
800μ
m=15.1±
0.2 . The image noise is thus ∼
1.6×
 higher for the larger ( 2.7×
 in the beam direction) sample.

**Fig. 4.**
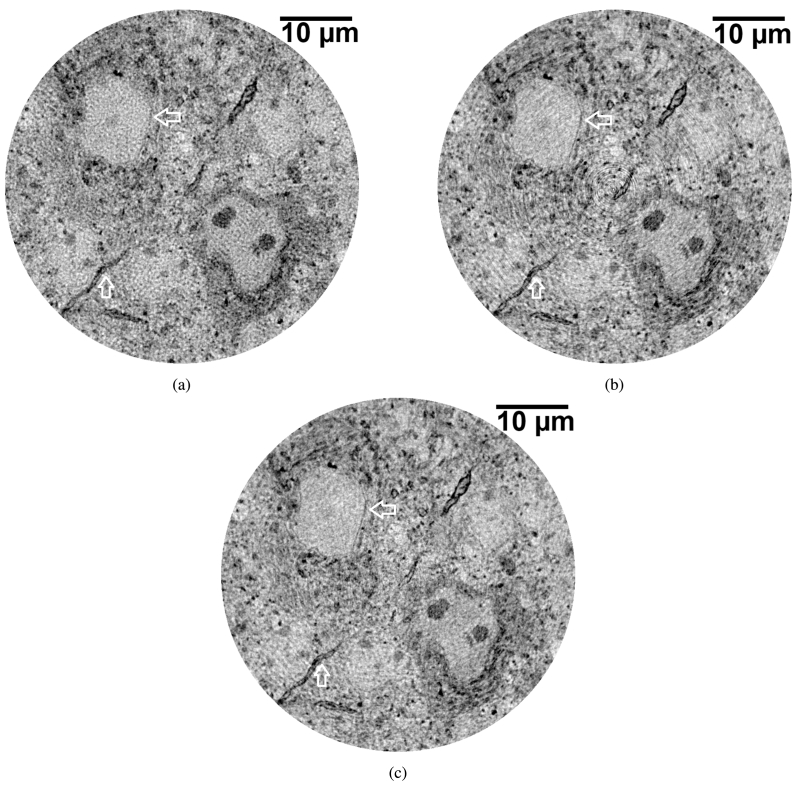
XNH images of mouse olfactory bulb tissue with a diameter of 800 μ m acquired using (a) step-by-step (SBS) scanning, (b) continuous scanning without ring correction, and (c) continuous scanning and reconstruction with Münch ring correction. The arrows indicate structures (cell body wall and myelin sheath) which appear more distinct (qualitatively) in continuous acquisition mode, with or without ring correction.

Figure 4 compares corresponding slices from images of the same sample, an 800 μ m diameter mouse cortex sample, acquired using SBS (Fig. 4(a)) and continuous acquisition modes without (Fig. 4(b)) and with (Fig. 4(c)) Münch post-processing ring correction. The noise and CNR were evaluated for each acquisition mode and are given in Table 5.

**Table 5.** Noise and CNR values of XNH images of 800 μ m diameter mouse cortex sample acquired using SBS and continuous modes.

Acquisition mode	σ	CNR
SBS	17.1 ± 0.3	5.0
Continuous	16.3 ± 0.3	6.2
Continuous (with Münch ring correction)	15.8 ± 0.3	5.9

The total time for SBS acquisition was 169 minutes, compared to 75 minutes for continuous acquisition.

### 3.3. Effect of holographic acquisition mode on image quality

**Fig. 5.**
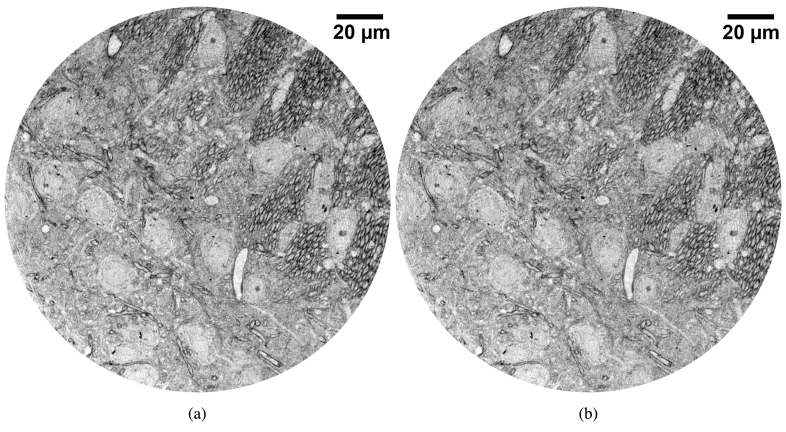
XNH images of mouse cortex tissue based on phase maps reconstructed using (a) four sample positions (D1, D2, D3 and D4 and (b) three sample positions (D1, D3 and D4).

Figure 5 shows slices from the dataset reconstructed using projections acquired at all four sample positions (5(a)) and at distances D1, D3 and D4.

The image noise for each reconstruction is σ
4distance=20.6±
0.4 for the complete dataset and σ
3distance=23.2±
0.4 for the incomplete dataset.

The acquisition time for the complete dataset was 51 minutes, compared to 38 minutes for the incomplete dataset.

## 4. Discussion

Synchrotron-based X-ray holographic nanotomography enables fast, non-destructive, high-resolution, large-scale 3D imaging of neural tissue without the need for serial sectioning. An integrated strategy, combining a detector upgrade as well as data acquisition and reconstruction developments, aiming at scaling up XNH for neural nanoimaging has been presented.

The detector upgrade consisted of replacing the previously installed FReLoN camera with the faster Ximea MX377 camera in order to accelerate data acquisition and thus increase sample throughput. The cameras were evaluated and compared in terms of their spatial resolution, measured using the Fourier Shell Correlation, and contrast to noise ratio between myelin and the tissue-free background. The spatial resolution measured for each camera regardless of exposure time (70 or 200 ms) were equal to within 3 nm (∼
1.3%
) . The absolute values, between 234 and 237 *μ *m, depend of course on the effective voxel size set by the choice of sample positions used in the holographic acquisition. As expected, the CNR was higher in each case for the longer exposure time (13.3 compared to 11.5 for the FReLoN camera, and 11.4 compared to 9.6 for the MX377 camera). The CNR of the MX377 camera is thus ∼
14−
17%
 lower than that of the FReLoN camera for the exposure times tested. The MX377 permits faster data collection than FReLoN camera, with a 3.6× faster acquisition time per frame for the 200 ms exposure, and 6.2× for the 70 ms exposure. Given the important decrease in acquisition time permitted by the MX377 camera, the lower CNR, which may in future be improved via other methods of noise reduction or a further upgrade to a higher grade CMOS sensor (for example, the scientific grade back-side illuminated variant), is considered a minor compromise. Defective pixels present in the engineering grade sensor are expected to generate ring artifacts in the reconstructed datasets, which are mitigated via random-motion displacements in step-by-step acquisition, or post-processing ring removal algorithms for continuous acquisition.

Accelerating data acquisition is not the only requirement for scaling up nanoimaging of neuronal tissues. Currently, sample size is limited in each dimension to several hundreds of *μ *m to minimise interior tomography effects, the "textured" image noise resulting from reconstructions using incomplete projections for volumes larger than the detector field-of-view (FOV). Upscaling neuroimaging requires imaging volumes of 1 mm^3^ and beyond to compete with the benchmark set by electron microscopy-based neural circuit reconstruction.

Image noise was quantified for two samples of the same specimen, with diameters of 300 *μ *m and 800 *μ *m, and was found to be 1.6×
 higher for the larger sample. The effect of different tomographic acquisition modes, step-by-step and continuous, on the image noise was also evaluated. Despite a slower acquisition time, step-by-step acquisition was previously favoured over continuous acquisition for XNH studies at ID16A for its compatibility with a ring suppression technique based on small, random displacements of the sample between successive projections.

Surprisingly, despite the visual presence of ring artifacts, the image acquired in continuous mode has both a lower noise level (16.3 compared to 17.1) and a higher contrast-to-noise ratio (6.2 compared to 5.0) than in SBS mode, which we hypothesise is due to the “blurring" of the textured noise resulting from interior tomography. The noise was further reduced (15.8) when ring correction [42] was applied during tomographic reconstruction, but no gain in contrast-to-noise ratio was observed. The unexpected benefit of noise reduction using continuous acquisition mode is in addition to an acquisition time which is two times faster than in SBS mode. As a result, this mode will be recommended for future XNH-based neural tissue studies. A comparative study using other ring correction algorithms, such as that proposed by [52], also implemented in Nabu, or novel machine learning-based methods [53,54], was outside the scope of this study but will be performed to optimise the balance between data acquisition and reconstruction time and image quality.

Finally, we investigated the effect of reducing the number of images used for the phase retrieval, by eliminating one of the sample positions at which tomographic acquisition is performed. Reducing the the amount of data acquired evidently reduces the data acquisition time, by 25%
 per excluded position. Somewhat expectedly, reducing the input to the phase retrieval algorithm leads to reduced image quality, observed as a 13%
 increase in image noise. This option may therefore not be appropriate for all neural tissue nanoimaging studies. It could, however, be implemented for studies with less stringent resolution requirements, such as reconstructing myelinated axons [55].

In general, combining the faster MX377 camera and continuous acquisition with post-processing ring correction has allowed a 60%
 reduction in acquisition time. For applications where a compromise in image quality can be made, further reductions can be made by acquiring data at fewer sample positions. The combined strategy currently allows for 2.5−
3×
 faster data acquisition than was previously possible, which could further be improved with faster sample positioning actuators. Previously, XNH scans of mouse posterior parietal cortex tissue with 100 nm voxels took ∼
4 hours, with sufficient spatial resolution to resolve and trace pyramidal cell apical dendrites [30]. Using the proposed combined strategy, the same scan would take as little as 80 minutes, and one could envisage the possibility of scanning entire sub-cortical white matter during a typical beamline experiment lasting 3-6 days.

XNH presents several advantages for large scale neural circuit mapping compared to the current gold standard, electron microscopy, which is extremely time consuming due to serial sectioning and sequential imaging, requiring extensive image processing and alignment. Additionally, EM, which relies on differential electron scattering, requires dense staining agents to enhance contrast. Samples included in this study were prepared using electron microscopy sample preparation protocols, including osmium tetroxide staining and EPON resin embedding, however, as XNH exploits phase contrast generated by differences in refractive index within biological tissues rather than absorption contrast, samples can be prepared without heavy metal staining [12]. Additionally, the ID16A endstation is cryogenically cooled, supporting imaging of frozen hydrated tissue samples and removing the need to embed samples in resin or paraffin.

A recent study [30] demonstrated that the spatial resolution of XNH (87 - 222 nm) and field-of-view of XNH allow dense reconstruction of individual neurons across millimeter-sized volumes. As such resolutions are insufficient for resolving synaptic connections, EM with sub-10 nm resolution remains the gold standard when synaptic resolution is required. The current spatial resolution of XNH is also far from the theoretical limits of hard X-rays. Hard X-ray ptychographic nanotomography is capable of achieving spatial resolutions lower than 40 nm and expected to be able to achieve resolutions as low as 20 nm [56], but is unable to match the speed or large field-of-view of XNH. Near-field ptychography is, however, being investigated as an approach for separating the probe from the sample in XNH data and allowing improved image quality and spatial resolution [57]. Furthermore, a self-supervised image restoration approach [54] was shown to improve spatial resolution by up to a factor two, and the contrast by up to a factor five in XNH data. The approach also supports the scaling up of XNH for neuronal tissue imaging, allowing two orders of magnitude increase in imaged volume for a given acquisition time and spatial resolution. With future implementation of such algorithms in the XNH reconstruction workflow, synaptic resolution with XNH might be achievable.

The main challenges related to XNH for neuronal tissue imaging are the computational cost for data reconstruction, particularly for the phase retrieval step, and sample deformation at the high imaging doses encountered in high resolution imaging. Image reconstruction, including phase retrieval, of data presented in this manuscript was carried out on the ESRF computing cluster. Phase retrieval currently requires approximately 2.5 hours per dataset. However, in order to share computing resources, the phase retrieval is not fully parallelised. Full parallelisation with the current computing resources available would reduce this time to approximately 10 minutes per dataset. New resins capable of resisting high radiation doses are being investigated [56], in addition to non-rigid stitching algorithms [58].

## 5. Conclusion

We have proposed a combined strategy that allows the upscaling of x-ray nanoimaging of neural tissues by allowing scans of larger samples- with a reduced acquisition time, whilst maintaining or even improving image quality. The strategy combines instrumentation development, or more specifically, a camera upgrade has resulted in a 10%
 faster acquisition with no impact on the spatial resolution, but albeit a small compromise (15% decrease) in contrast-to-noise ratio, which may be alleviated via a further upgrade to a higher grade CMOS sensor or implementation of noise reduction methods at the image processing stage. Continuous tomographic acquisition allows for a further 2×
 reduction in data acquisition time compared to step-by-step acquisition and, although incompatible with random motion-based ring artifact suppression, results in reduced image noise with further reductions reported when post-processing ring suppression is applied. Phase retrieval using fewer sample positions allows for an additional 25% decrease in time for each position excluded, however, this reduction in acquisition time comes at the expense of increased noise which may be acceptable for certain applications. Overall, the acquisition time has been reduced by 2.5−
3×
 , making it possible to cover larger volumes and access data which was previously out of reach.

## Data Availability

Data underlying the results presented in this paper are not publicly available at this time but may be obtained from the authors upon reasonable request.
